# Increasing the efficiency of cone-beam CT based delta-radiomics using automated contours to predict radiotherapy-related toxicities in prostate cancer

**DOI:** 10.1038/s41598-024-60281-6

**Published:** 2024-04-26

**Authors:** Rodrigo Delgadillo, Anthony M. Deana, John C. Ford, Matthew T. Studenski, Kyle R. Padgett, Matthew C. Abramowitz, Alan Dal Pra, Benjamin O. Spieler, Nesrin Dogan

**Affiliations:** 1 Department of Radiation Oncology, University of Miami Miller School of Medicine, 1475 NW 12Th Ave, Miami, FL 33136 USA; 2 Varian Medical Systems, Advanced Oncology Solutions, Avon, IN USA

**Keywords:** Predictive markers, Prostate cancer, Cancer imaging, Tumour biomarkers

## Abstract

Extracting longitudinal image quantitative data, known as delta-radiomics, has the potential to capture changes in a patient’s anatomy throughout the course of radiation treatment for prostate cancer. Some of the major challenges of delta-radiomics studies are contouring the structures for individual fractions and accruing patients’ data in an efficient manner. The manual contouring process is often time consuming and would limit the efficiency of accruing larger sample sizes for future studies. The problem is amplified because the contours are often made by highly trained radiation oncologists with limited time to dedicate to research studies of this nature. This work compares the use of automated prostate contours generated using a deformable image-based algorithm to make predictive models of genitourinary and changes in total international prostate symptom score in comparison to manually contours for a cohort of fifty patients. Area under the curve of manual and automated models were compared using the Delong test. This study demonstrated that the delta-radiomics models were similar for both automated and manual delta-radiomics models.

## Introduction

One of the major challenges of longitudinal delta-radiomics studies is contouring the individual fractions for radiomic feature extraction. The problem is exemplified in prostate cancer (PCa) radiotherapy (RT) where a typical treatment is forty fractions. A time inefficient approach is to manually contour each individual fraction to select the region of interest (ROI) for radiomic analysis. The time it takes to contour the patients is compounded by the fact that the individuals that are qualified to delineate the contours are highly trained, typically physicians, and are busy with other clinical duties. In addition, radiomics studies are often limited by the number of patients that can be included in such studies. Automated contours of the ROI could greatly increase the efficiency and feasibility of implementing such studies^1,2^.

Though prostate cancer is the second-leading cause of cancer-related mortality in the United States (US)^3^, the number of PCa survivors is 3.1 million in the US^4^. Consequently, with so many survivors, a significant portion of men can be impacted by acute and late side effects of PCa RT^4^. A previous pilot study used daily Cone Beam CT (CBCT) images to generate delta-radiomics models to predict acute and sub-acute genitourinary (GU) toxicity and change in total international prostate symptom score (IPSS) in PCa RT patients^5^. However, that work used manual contours. Alternative approaches should be considered to efficiently increase the number of patients. If the models produced using automated contours are of similar performance to the manual contours, future studies could accumulate more patients more efficiently.

The purpose of this study is to test the effectiveness of automated contours of the prostate to produce delta-radiomics models to predict acute and sub-acute GU and change in IPSS in PCa RT patients using a deformable image registration (DIR) algorithm. This work combines methodology and lessons learned from our previous work^1,5,6^. Schmidt et al.^1^ assessed CT to CBCT contour mapping for radiomic feature analysis in prostate cancer by analyzing correlation and mean absolute difference of radiomics features extracted from manual and automated contours of daily CBCT images. Schmidt et al.^1^ also showed that automated and manual contours of the CBCT prostate contours matched well using metrics described in American Association of Physicists in Medicine (AAPM) Task Group (TG)-132^7^ using quantitative metrics such as Dice similarity coefficient (DSC), mean distance-to-agreement (MDA), difference in center-of-mass position (ΔCM), and difference in volume(ΔVol). The current study moves on to the next step by analyzing the capability of automated contours to produce predictive delta-radiomics models using the methods described in a previous work^5^, and compares the automated-contour model performance and the manual-contour model performance. If the automated-contour model performance is of similar performance to the manual-contours, this may indicate that DIR based algorithms could be of use in future delta-radiomics to improve efficiency of data collection and analysis.

## Materials and methods

### Patient selection

Fifty PCa patients who were treated with Volumetric Modulated Arc Therapy (VMAT) were retroactively selected from an internal review board (IRB)-approved protocol. The ethical approval for this study was obtained from the University of Miami Institutional Review Board (IRB). Written informed consent was obtained from all patients in this study. The data was retrospectively collected and analyzed. All methods undertaken in this work were carried out in accordance with the relevant guidelines and regulations. The median age of the patients was 50 years old (range 50–87). Other Patient characteristics including the Gleason Score, tumor stage, and hormonal use are shown in Supplementary Table S1. To increase radiomic feature consistency and reduce variability, several patient selection criteria were established for this study. Only PCa patients treated with VMAT on TrueBeam accelerator (Varian Medical Systems, Palo Alto, CA) with daily CBCT image guided radiotherapy were included in this study. Raw projection data of each CBCT was exported from the treatment machines and later reconstructed using image reconstruction software to maintain consistent reconstruction parameters for all the images. More details on image reconstruction are described in section "Image parameters". Patients with intrapelvic metal prostheses produced metal streaking artifacts that resulted in poor radiomic feature quality and were hence excluded from the study. Patients with body mass index (BMI) > 40 were excluded due to x-ray hyper attenuation. Patients were treated with a variety of dose fractionation schedules and shown in Supplementary Table S2. Most of the patients were treated with one of two dose fractionation schedules: 80 Gy in 40 fractions (26 patients) and 70.2 Gy in 26 fractions (13 patients). The remaining patients were treated on other dose fractionation schedules. PCa patients treated with Stereotactic Body Radiotherapy (SBRT) were not considered in this study. A biologically effective dose (BED) based averaging method was used to account for the different dose fractionations and is described in more detail in section "BED-based Binning of DRF".

### Image parameters

Image quality and other characteristics such as voxel size and reconstruction parameters have been shown to affect radiomic features^6,8–12^. Consequently, this study was designed to minimize variables in the study where possible by keeping the image characteristics consistent. The image size (512 × 512 pixels), pixel size (0.9 mm), slice thickness (2 mm), FOV (465 mm), tube voltage (125 kVp), and tube current–time product (1073–1074 mAs) were consistent for all CBCT scans.

**Figure 1:**
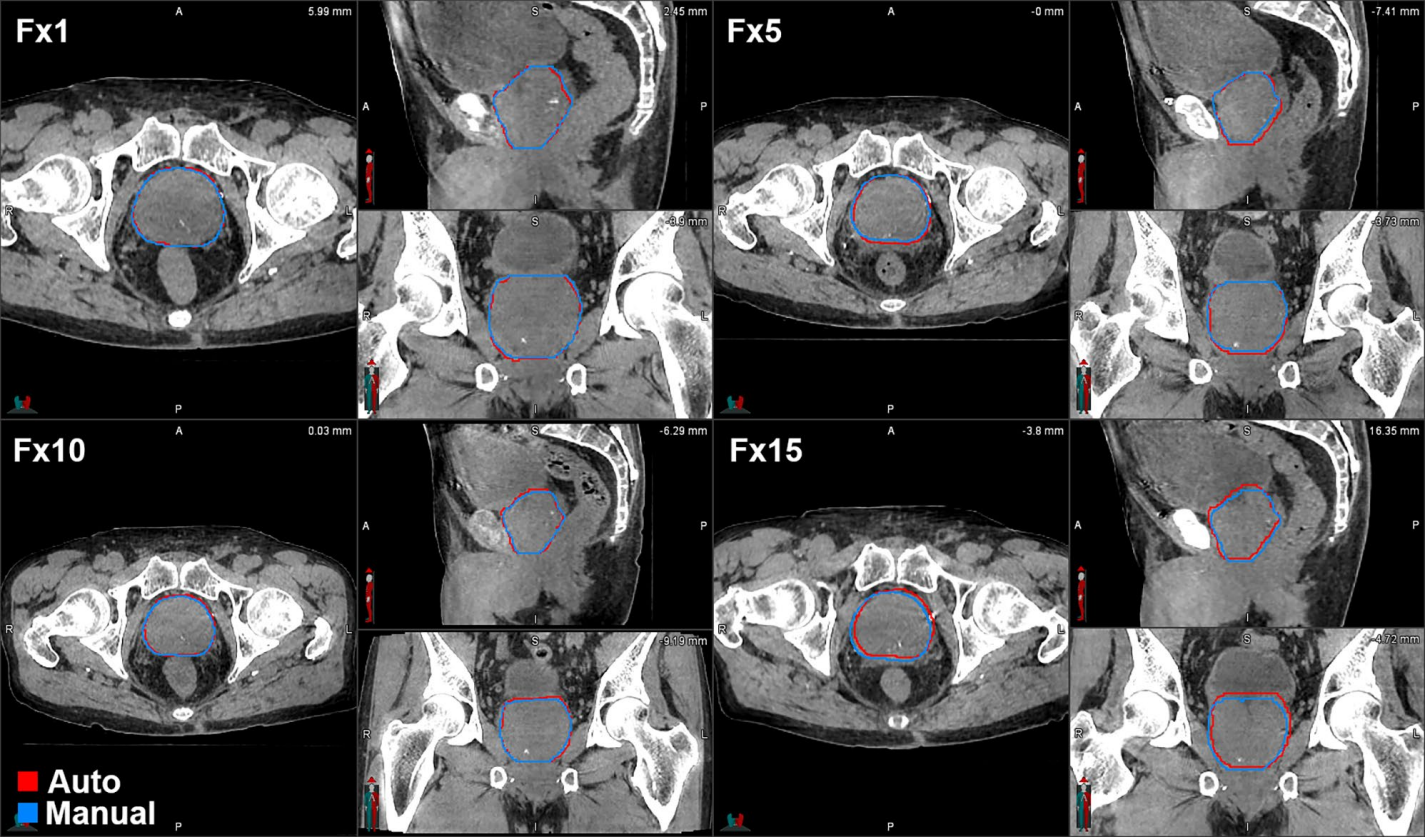
Example CBCT images of a prostate patient for different fractions during radiotherapy treatment. Automated prostate contours are shown in red and manual contours in blue. The images shown here were reconstructed using iterative reconstruction algorithm.

A research image reconstruction software (iTools, Varian Medical Systems, Palo Alto, CA) was used to generate either standard convolution CBCT (sCBCT) or iterative CBCT (iCBCT) image sets for each patient. An example patient’s CBCT images for different fractions along the course of radiotherapy are shown in Fig. 1. Daily CBCT raw projections were reconstructed utilizing the typical reconstruction parameters used in the clinic. The two most used clinical reconstruction parameters in our clinic use standard convolution filter for both iCBCT and sCBCT. iCBCT refers to an iterative reconstruction algorithm that estimates the scatter in the raw projection images with the aim to correct for the scatter in the final reconstruction through iterative attempts and by solving the linear Boltzmann transport equation^13^ iCBCT images have a parameter known as noise suppression that controls the level at which the iterative process reduces scatter noise. A medium noise suppression on iCBCT was considered for this study because it represents the default clinical value.

Images were processed prior to radiomic feature extraction. The Image Biomarker Standardization Initiative (IBSI) defines image processing as procedures such as interpolation, range-re-segmentation, discretization (quantization), and image filtering^14^. For this study, the aim was to keep image processing as basic as possible to highlight the differences between the automated and manual contour models. Using IBSI notation, images were processed using intensity discretization with fixed bin number^14^. Previous CBCT-based radiomics studies of PCa have shown that discretization bins equal to 256, also known as 8 bit, to be repeatable and reproducible for radiomic feature extraction and produce well performing models^14^. For this reason, images were discretized using 256 discretization bins in this study.

### Prostate contours

Radiation Oncologists who specialize in treatment of PCa patients contoured the prostate on the planning CT (pCT) with the aid of Magnetic Resonance Imaging (MRI) for each patient. The daily CBCTs (both sCBCT and iCBCT) were rigidly registered during daily patient setup. Using the daily setup rigid registrations, the pCT prostate contours were transferred to the daily CBCTs. However, it was noticed that the prostate may change in volume or shape between the pCT and daily CBCT. Moreover, registration errors may produce other inconsistencies in the prostate contour. For this reason, a radiation oncologist reviewed the daily CBCT prostate contours and corrected the contours when needed directly on the CBCTs utilizing imaging software (MIM, ver. 6.8.1, MIM Software Inc., Cleveland, OH). In this work, radiomic models derived from the manual contours are referred to as manual models for conciseness. Similarly, models derived from the automated contours are referred to as automated models. Automated daily prostate contours were generated using a commercial DIR software (Velocity Advanced Imaging, ver. 4.1, Varian Medical Systems, Palo Alto, CA) with the method described in Schmidt et al.^1^. The DIR algorithm used here was the B-spline algorithm, which is an intensity-based algorithm based on the Mattes formulation of mutual information^15–17^.

Gold fiducial artifacts present in PCa patients are a source of imaging inconsistencies. An algorithm described in detail in Delgadillo et al.^6^ was used to produce masks to remove the gold fiducial artifacts within the prostate. On average, 18% of the prostate volume is lost due to the presence of gold fiducial artifacts in PCa patients^5^. Though some information is lost due to the presence of gold fiducial artifacts, the algorithm allows for the use of radiomics of multiple slices for analysis instead of other works which are limited to one slice radiomic^18^. By using a greater portion of the prostate volume, we may increase the chances of capturing changes that reflect the clinical outcomes.

As a form of quality assurance, the DSC was calculated between the automated and manual contours of all fractions of the CBCT images in the dataset, a total of 1711 individual fractions. DSC is statistical metric for comparing the volumetric overlap of two contours on registered images overlap^19^. The DSC between the automated and manual CBCT prostate contours was found to be 0.89 ± 0.12 (avg ± td). AAPM TG-132 states that contouring uncertainty within 0.8–0.9 is acceptable^7^. This work is an updated result for a previous work that estimated similar metrics for 28 patients^1^.

### Delta-radiomic features

This study focused on radiomic features from commonly used classes. Though many more radiomic features are possible, previous studies have shown that many radiomic features are intercorrelated^11,12^ and thus not quite different from one another. Five classes of radiomic features were considered, including: gray-level co-occurrence matrices (GLCM), Neighborhood Gray-Tone Difference Matrix (NGTDM), gray-level run length matrices (GLRLM). A full list of the forty-two radiomics features and 5 features classes considered in this study are shown in Supplementary Table S3 along with the IBSI code equivalent. Volume-normalized versions of NGTDM Strength, NGTDM Busyness, NGTDM Coarseness, GLSZM GLN, GLRLM GLN, and GLRLM RLN were also considered to reduced voxel size dependence of radiomic features following volume normalization methods described by Shafiq-ul-Hassan et al.^9^ and Fave et al.^8^

The delta-radiomic feature (DRF) was defined as:

$$DRF=\frac{R{F}_{N}-R{F}_{1}}{|R{F}_{1}|}$$

where $R{F}_{N}$ is the radiomic feature value of the Nth fraction and $R{F}_{1}$ is the radiomic feature value of the first fraction.

As a form of quality assurance, the radiomics features extracted from the prostate of the pCT and the first fraction (FX1) CBCT for fifty patients were compared using a spearman correlation. The Benjamini–Hochberg adjustment was applied to p-values to account for multiple comparisons^20^. An alpha of 0.05 was chosen to set the significance threshold for the adjusted p. This work represents an update to the results from a previous work that had only utilized 20 patients^6^.

### Clinical endpoints

**Figure 2:**
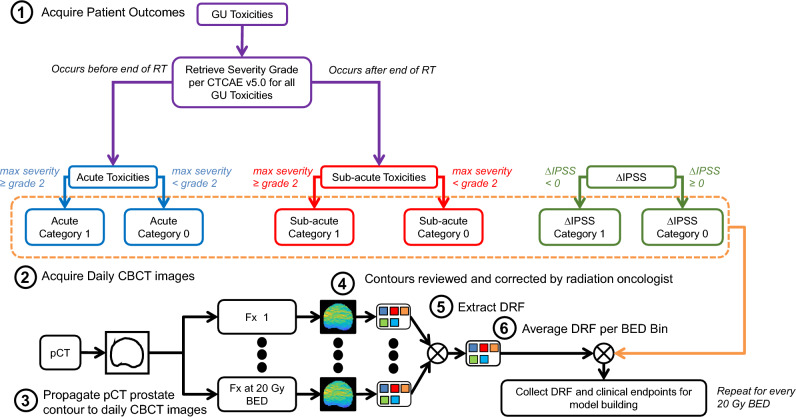
Visual representation of categorization of clinical endpoints and extraction of delta-radiomic features (DRF) from daily prostate CBCT images for analysis and model building.

Clinical Endpoints considered include the GU adverse events graded per CTCAE v5.0 and total IPSS^21,22^. GU toxicities considered were frequency, nocturia, dysuria, urgency, urinary obstructive symptoms, and incontinence. GU toxicities were further categorized based on the timing of the occurrence. Acute GU toxicities were toxicities that occurred before the end of RT. Sub-acute GU toxicities were toxicities that occurred after RT. Changes to the IPSS were used to describe changes in the patient’s urinary quality of life due to RTRT. The change in IPSS ($\Delta IPSS$) from before RT and after RT was used a clinical endpoint for model building. A visual representation of the categorization of the clinical endpoints for model building is shown in Fig. 2.

### BED-based Binning of DRF

To account for the different dose fraction schedules of the patients in this study, daily DRF were binned and averaged into 20 Gy BED bins. 20 Gy BED bins were chosen because it represents one week of treatment. The BED was calculated for each fraction using $\alpha /\beta$ = 3 to account for dose delivered to the prostate^23^. Averaging the DRF into BED bins improved signal to noise, reduced complexity of the analysis, and allowed the use of different dose fractionation schedules.

### Model building

DRF models of the previously discussed clinical endpoint were generated using logistic regression for every BED bin. The model building and analysis workflow follow the procedure described in a previous work^5^. For the sake of completeness, the workflow is summarized below.

The model building workflow starts with feature selection to identify radiomic features that are important to the models using random forest^24,25^. Feature selection was considered independently for acute GU toxicity, sub-acute GU toxicity, and $\Delta IPSS$. The selected DRF were kept consistent by considering all BED bins together during the feature selection phase. The DRF were ranked according to feature importance using the GINI index, which is calculated by first accumulating the changes in the risk due to splits on every predictor and by then dividing the sum of the number of branch nodes^26^. As discussed earlier, DRF may be intercorrelated. To reduce the impact of the intercorrelation and provide more unique DRF in the feature selection phase, highly intercorrelated (correlation > 0.8) radiomic features were filtered from the feature space while keeping only the most important radiomic features of a correlated group. The ranking of the radiomic features using the GINI is shown in Supplemental Figs. S1–S3. This results in a smaller feature space with more unique features to consider. Up to the top seven important and non-correlated DRFs were selected for model building. For each pair, the automated and manual models were constrained to use the same selected DRF for model building. This was to reduce the impact of feature selection in comparison between the automated and manual models. A summary of the selected DRF per clinical endpoint is shown in Table 1.

**Table 1:** Summary of the top DRF found after the feature selection phase organized by clinical endpoint and reconstruction and pre-processing method. Uniform refers to intensity discretization with fixed bin number.

Clinical Endpoint	Reconstruction and Pre-processing Method	N	Selected features
Acute	iCBCT Standard Medium Uniform	4	GLSZM LZHGE, NGTDM Coarseness-VN, NGTDM Strength, Prostate Volume
Acute	sCBCT Standard Uniform	6	GLRLM GLN, GLRLM GLN-VN, GLSZM ZSN, GLSZM ZSV, Global Kurtosis, Prostate Volume
Sub-Acute	iCBCT Standard Medium Uniform	5	GLSZM GLN, GLSZM LZHGE, GLSZM LZLGE, GLSZM ZSV, NGTDM Coarseness
Sub-Acute	sCBCT Standard Uniform	4	GLRLM RLN, GLSZM ZSN, Global Skewness, NGTDM Coarseness-VN
∆IPSS	iCBCT Standard Medium Uniform	4	GLRLM RLN, GLSZM SZLGE, GLSZM ZP, IPSS Baseline
∆IPSS	sCBCT Standard Uniform	6	GLCM Correlation, GLRLM RLN, GLSZM GLN-VN, IPSS Baseline, NGTDM Coarseness, NGTDM Contrast

After feature selection, the clinical endpoints were modeled using a logistic regression using a leave-one-out cross validation approach with one thousand bootstrapping iterations. The results of the feature selection are shown in Table 1. The leave-one-out approach was used due to sample size of fifty being too small to both have a training set and an independent validation set. It is the hope that the results of this work will allow for future works to overcome small size by making the process of delta-radiomic studies more efficient.

To compare delta-radiomics to snapshot-radiomics, models were built using radiomic features extracted from prostate contours on pCTs for all the patients. Then, the AUC of pCT radiomics models were compared to CBCT based models for the Sub-Acute GUT and ∆IPSS models. The Acute model could be calculated for pCT since there are no longitudinal simulation CTs recorded during treatment. The features used in the models were kept consistent between the CBCT and pCT. Consequently, four pCT radiomic models were created using the bootstrapping approach described previously for CBCT.

### Data analysis

All data analysis, which included the model building, was performed using a statistics and machine learning toolbox from scientific computation software (MATLAB, ver. 2020b, Math-Works Inc., Natick, MA). The area under the curve (AUC) and their confidence intervals and receiving operating characteristic curves (ROC) were calculated from the result of the bootstrap analysis. The Delong test^27^ (threshold p < 0.05) was used to test the similarity between the AUC manual and automated models. Delong’s algorithm is one of the most widely used algorithm to compare the AUC of two or more ROCs^28^.

## Results

**Figure 3:**
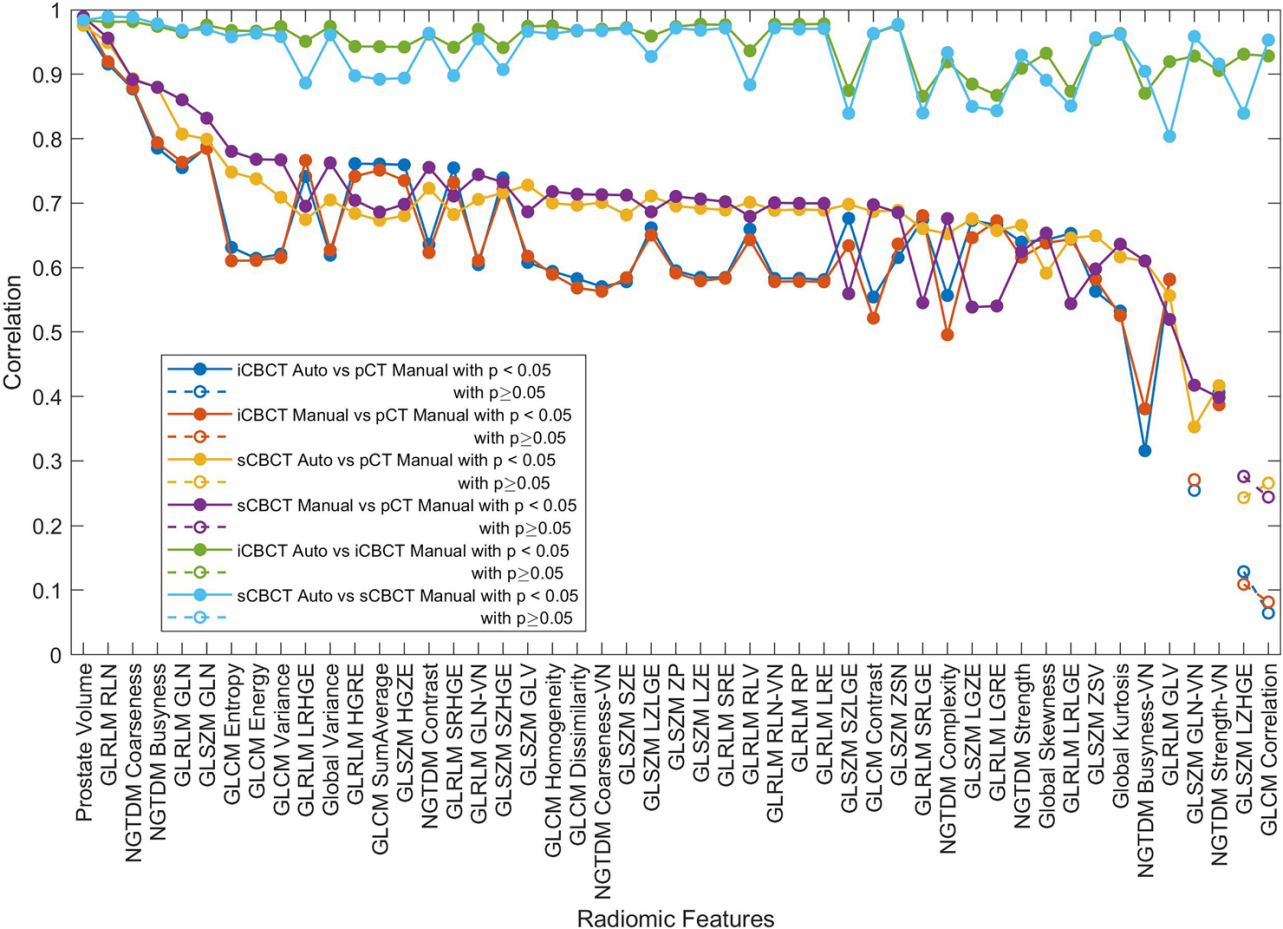
Spearman correlation of radiomic features extracted from the automated and manual contour of the prostate for the FX1 iCBCT vs pCT, FX1 sCBCT vs pCT. The automated and manual radiomic features were also compared for both FX1 iCBCT and FX1 sCBCT reconstructions. Correlations were performed with a sample size of fifty patients.

**Figure 4:**
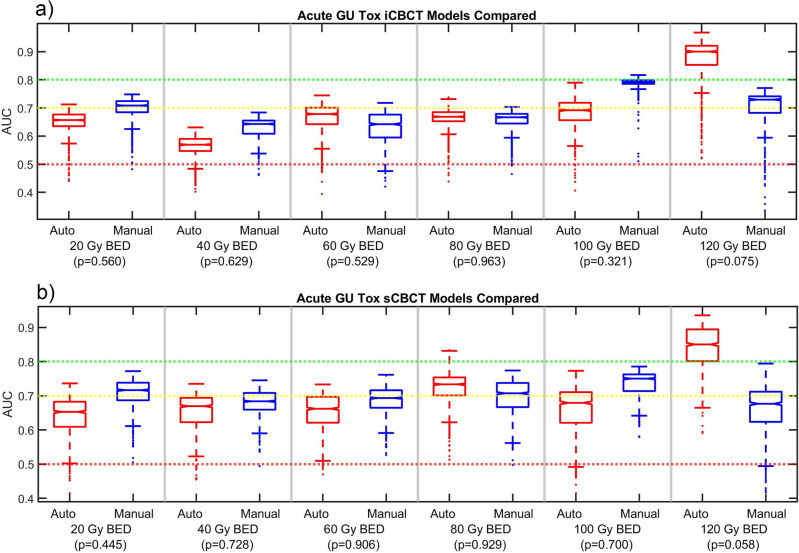
AUC data for the Acute GU Tox (**a**) iCBCT and (**b**) sCBCT delta-radiomic models. Delong p-values between Auto and Manual AUC shown in parenthesis.

**Figure 5:**
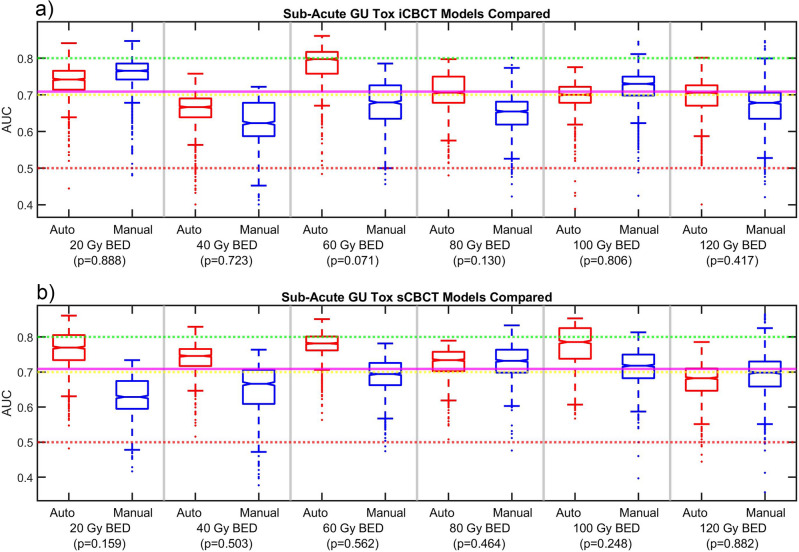
AUC data for the Sub-Acute GU Tox (**a**) iCBCT and (**b**) sCBCT delta-radiomic models. Delong p-values between Auto and Manual AUC shown in parenthesis. The mean pCT model AUC is shown as a magenta line across the figure.

**Figure 6:**
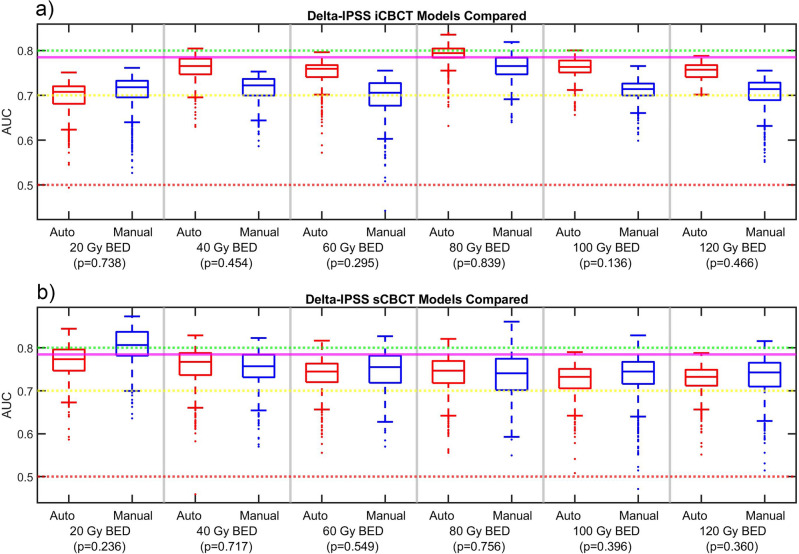
AUC data for the ∆IPSS (**a**) iCBCT and (**b**) sCBCT delta-radiomic models. Delong p-values between Auto and Manual AUC shown in parenthesis. The mean pCT model AUC is shown as a magenta line across the figure.

The results are summarized in Figs. 3, 4, 5 and 6. Figure 3 shows that the automated and manual radiomic features have statistically significant correlations for all but two radiomic features between the pCT and the FX1 CBCT. Typically, the sCBCT radiomic features correlate more strongly with the pCT that iCBCT radiomic features. When compared automated and manual radiomic features for CBCT (either sCBCT or iCBCT), the radiomic features correlate highly indicating good agreement between the automated and manual contours. This result is consistent with the DSC values with 0.89 ± 0.12 (avg ± td) between the automated and manual contours of CBCT.

A summary of the top DRF found after the feature selection phase organized by clinical endpoint and reconstruction and pre-processing method is shown in Table 1. For all BED bins, reconstruction methods, and clinical endpoints considered, the automated and manual models produced AUC with p > 0.05. This indicated that the automated and manual models were of similar performance. In other words, automated and manual model AUC were not significantly different. AUC between 0.7 (yellow line) and 0.8 (green line) is considered to be moderately performing, above 0.8 strongly performing, and below 0.7 weakly performing. AUC below 0.5 (red line) indicates model performance is no better than random chance. The pCT model mean AUC was also included in the AUC figure when appropriate.

Delong p values were mostly well beyond the threshold for significance. However, some automated vs manual model comparisons were just above the threshold. The p-value for the acute GU toxicity for 120 Gy BED was 0.075 for iCBCT and 0.058 for sCBCT, as shown in Fig. 4. For the 120 Gy BED bin of acute GU toxicity, the automated model distribution was higher than the manual model distribution. The p-value for sub-acute GU toxicity for 60 Gy BED bin was 0.071 for iCBCT, as shown in Fig. 5. Consequently, though most models were far beyond the threshold for a statistically significant difference, for the three exceptions that had p-values between 0.058 and 0.075, the delta-radiomic model using automatically segmented contours outperformed the manually contoured.

The DRF models of acute GU toxicity were performing weakly for both the iCBCT and sCBCT models in Fig. 4. Though no statistical significance differences were found, often the manual models had a median AUC slightly higher than the automated models of acute GU toxicity. However, the top performing acute GU toxicity models occurred for the automated models at the 120 GY BED bin.

For the sub-acute GU toxicity models, the sCBCT models have median AUC between 0.75 to 0.92 across all the BED bins, while the iCBCT models waver between median AUC 0.65 to 0.75, other than the 120 Gy BED bin that has 0.85 median AUC at 120 Gy BED for the automated model. The pCT model of sub-acute GU toxicity had a mean AUC of 0.71. The pCT model of sub-acute GU toxicity often performed similarly to the varied BED time points of the iCBCT models, as shown in Fig. 5. The iCBCT models performed better than the pCT sub-acute models for the 20 Gy and 60 Gy BED models. The automated sCBCT models performed better than the pCT models of sub-acute toxicity for all but the last 120 Gy BED model.

The ∆IPSS models shown in Fig. 6 perform the most similarly between the automated and manual models. The median AUC is more consistently greater than 0.7 for ∆IPSS than the other clinical endpoints considered in this work. The pCT model of ∆IPSS had a median AUC of 0.78. Consequently, the pCT model performed better than the CBCT models for the majority of BED time points, as shown in Fig. 6. However, the best iCBCT model at 80 Gy BED performed better than the pCT model and the best sCBCT model at 20 Gy BED performed better than the pCT model.

## Discussion

Delta-radiomics studies have the potential of capturing changes in the patient’s anatomy that could inform us of the effectiveness of RT and potentially design mitigation strategies to overcome some of the negative effects of RT. This study focused on GU toxicities and ∆IPSS as clinical endpoints due to relatively high occurrence in PCa patients undergoing RT. Moreover, these clinical endpoints occur in the short-term. Meaning that the study could be designed to model negative effects without waiting long after treatment. Other clinical endpoints with a small percentage of occurrence would require many more patients to build a meaningful model. For, example five-year survival of prostate cancer is 97%^29^ and would take a large sample size to properly model the other 3% .

A literature search reveals that few CT-based studies have performed in a pre-treatment setting for prostate cancer^30–32^. Tanadini-Lang et al. used morphological, statistical, and transform-based radiomics to predict Gleason score and found AUC between 0.77 and 0.8. Osman et al. used statistical and transform-based radiomic features to predict risk group using tumor grade, GS, and PSA and found AUC between 0.9 and 1.0. Thus, these pre-treatment CT-based studies tend to perform very well. To our knowledge, however, no pre-treatment study was found to predict GU toxicity or ∆IPSS.

One may wonder whether the effort of performing deltaradiomics studies is worth it comparison to snapshot radiomics, such as using pCT. Considerable effort goes into contouring all the fractions and extracting the radiomic features from those fractions in deltaradiomics studies such as this one. Moreover, the image quality of the pCT images is higher than the CBCT images. For this reason, models of the sub-acute GU toxicity and ΔIPSS were built using the pCT and compared to the CBCT deltaradiomics models. The pCT models performed better in some cases and worse in other cases compared to the CBCT models. The three major variables were the reconstruction algorithm used for the CBCT, the patient outcome considered, and the contouring type, i.e., automated vs manual. The sCBCT models often performed better than iCBCT models. The correlation between the CBCT and pCT radiomics features, shown in Fig. 3, often showed that sCBCT correlated better than iCBCT with pCT. This may indicate that the sCBCT radiomic features are more like the pCT radiomic features and thus produce better quality deltaradiomics models more on par with the pCT models. The patient outcomes that were modeled as resulted in different performances indicating that some clinical outcomes are more suited to deltaradiomics studies than others. In this case, the sub-acute GU toxicity models were more suited to deltaradiomics than the ΔIPSS models. The reason is not yet understood and could be explored in future research.

Automated segmentation has become increasingly studied because manual segmentation is time-consuming task, has significant inter-observer and intra-observer variability and automated segmentation could be valuable to adaptive therapy, radiomic, and dosimetric analysis. The most popular auto-segmentation techniques can be clustered into two categories including registration techniques and deep learning techniques^33^. Registration techniques are considered a more traditional approach, but significant improvements have been made in deep learning techniques in recent years^33^. A future study could investigate deep learning techniques and their applicability to delta-radiomic analysis, and these new deep learning techniques may even have higher accuracy. However, this is an ongoing area of research, so it is too early to know for certain without conducting another study. A literature review of prostate-based studies automatic segmentation reveals MRI^34,35^, ultrasound^36^, CT^37,38^, and CBCT^37–41^ have been considered. However, to our knowledge, no other study of PCa has evaluated performance of automated CBCT contours of the prostate for delta-radiomics models. Moazzezi et al. 2021 has evaluated the CBCT guided daily adaptive radiotherapy (Ethos, Varian Medical Systems, Palo Alto, CA) using unedited CBCT auto-segmentation and found that 96% of fractions required auto-segmentation edits, although the corrections were generally minor^39^. Thus, though most automated studies had satisfactory results they were far from perfect. As previously mentioned, the automated workflow described in Schmidt et al.^1^ was implemented for this current work and provided support evidence that automated contours may be applicable for CBCT-based radiomics studies of prostate cancer. Schmidt et al.^1^ also considered contour comparisons following recommendations from AAPM TG-132^7^, which provides recommendations on the use of image registration and fusion algorithms and provides quantitative methods of evaluating DIR accuracy. They found that the mean DSC, MDA, ΔCM and ΔVol between the auto and manual prostate contours were 0.90 ± 0.04, 1.81 ± 0.47 mm, 2.17 ± 1.26 mm and 5.1 ± 4.1% respectively^1^. Nearly 95% of DIRs were within tolerance recommendations of TG-132^1^. Then, this study posits the idea that radiomic features may be robust to minor contour changes and thus suitable for delta-radiomic analysis. The updated DSC results for the fifty patients in this study agree with the results from the previous study.

For all clinical endpoints considered in this study, both the automated and manual contours produced radiomic models of similar performance. In three cases, the Delong p-values were slightly above 0.05. it may be possible to find statistically significant differences with more patients. However, if these three cases were found to have a p less than 0.05, it would speak more in favor of the models created using automatically generated contours than against them. Interestingly, the automated models performed better than the manual models in these three cases. We do not fully understand why automated models may be better than manual models in some cases. However, automated contours may have some advantage over manual contours. One benefit of the automated contours is that there is less user-to-user variability in contours. Another advantage is that automated contours is that the user does not have to be a contouring expert. A user with limited knowledge of anatomy but basic computer skills could generate automated contours. In some cases, like in Fig. 5b, the automated models often were performing better than the manual models. Though care should be taken to make any definitive statements since the p-value was not significant. A future work could investigate comparing automated models in comparison the manual models based on different users to speak more to effect of user variability.

Given that the automated models performed on par with the manual models, this study indicates that automated models may be useful for future delta-radiomics studies. Automated techniques to contour the prostate may allow researchers to acquire more patients. This could enable studying lower occurring clinical endpoints like recurrence that would not be feasible for low sample size. For large scale multi-institutional projects, researchers of radiomics are faced with many challenges like different treatment protocols, patient populations, treatment modalities, and variability in imaging characteristics and modalities. Thus, facing the challenges directly will allow for these future works to progress. In this work, we attempted to take on the contouring portion of the challenges.

## Conclusion

This study demonstrated that the differences in delta-radiomic model performance is not statistically significant between manually or automatically contoured prostate volumes on daily CBCT. The results are consistent for both the sCBCT and iCBCT based models. Thus, the use of DIR to automatically generate prostate contours is a suitable replacement for manual contouring for CBCT-based delta-radiomics. This will aid future studies in accumulating larger sample sizes.

### Supplementary Information

Supplementary Information.

## Data Availability

The datasets generated and/or analyzed during the current study are not publicly available due to still being collected as part of an ongoing clinical trial. Clinical trial relevant data will be made public upon completion of accrual and publication of the primary endpoint. Contact the corresponding author, N.D, to request data from this study.
