# Supplementary material for: Increasing the efficiency of cone-beam CT based delta-radiomics using automated contours to predict radiotherapy-related toxicities in prostate cancer
Source: Sci Rep. 2024 Apr 26;14:9563. doi: 10.1038/s41598-024-60281-6 (PMC11053114; doi:10.1038/s41598-024-60281-6)
Supplement: Supplementary file 1 — Supplementary Information. [file 41598_2024_60281_MOESM1_ESM.docx]

Supplementary Information

# Supplementary Tables

**Supplementary Table S1**. Patient characteristics.

| Characteristic | Number of Patients | Percent of Patients (%) |
| --- | --- | --- |
| Gleason Score |  |  |
| 3+3 | 8 | 16 |
| 3+4 | 14 | 28 |
| 4+3 | 7 | 14 |
| 4+4 | 4 | 8 |
| 4+5 | 14 | 28 |
| 5+4 | 1 | 2 |
| 5+5 | 1 | 2 |
| Missing | 1 | 2 |
| Tumor Stage |  |  |
| T1c | 18 | 36 |
| T2a | 3 | 6 |
| T2b | 8 | 16 |
| T2c | 4 | 8 |
| T3a | 9 | 18 |
| T3b | 2 | 4 |
| Missing | 6 | 12 |
| Hormonal Use |  |  |
| Used | 37 | 74 |
| None | 13 | 26 |

**Supplementary Table S2**. Patient dose fractionation schedules.

| Total dose (Gy) | Total fractions | Number of Patients | Percent of Patients (%) |
| --- | --- | --- | --- |
| 70.2 | 26 | 13 | 26 |
| 74 | 37 | 1 | 2 |
| 76 | 38 | 5 | 10 |
| 80 | 40 | 26 | 52 |
| 81.4 | 37 | 1 | 2 |
| 86 | 40 | 1 | 2 |
| 91.2 | 38 | 3 | 6 |

**Supplementary Table S3.** Forty-two radiomic features were considered in this study. IBSI codes listed in brackets.

| **Feature Class** | **Description** | **Features** |
| --- | --- | --- |
| Gray-level Co-occurrence Matrices  (GLCM) [LFYI] | GLCM feature used voxel displacements of 1 to quantify the frequency of a pattern of two intensities occurring together throughout an image. | Contrast [ACUI] Correlation [NI2N] Dissimilarity [8S9J] Energy [8ZQL] Entropy [TU9B] Homogeneity [IB1Z] Sum Average [ZGXS] Variance [UR99] |
| Gray-level Run Length Matrices  (GLRLM) [TP0I] | GLRLM features encode for different run lengths of connected isotone voxels. Runs are computed in 13 directions of three-dimensional space. | Gray-level Non-Uniformity (GLN) [R5YN] Gray-level Variance (GLV) [8CE5] High Gray-level Run Emphasis (HGRE) [G3QZ] Long Run Emphasis (LRE) [W4KF] Long Run High Gray-level Emphasis (LRHGE) [3KUM] Long Run Low Gray-level Emphasis (LRLGE) [IVPO] Low Gray-level Run Emphasis (LGRE) [V3SW] Run Length Variance (RLV) [SXLW] Run Percentage (RP) [9ZK5] Run-Length Non-Uniformity (RLN) [W92Y] Short Run Emphasis (SRE) [220V] Short Run High Gray-level Emphasis (SRHGE) [GD3A] Short Run Low Gray-level Emphasis (SRLGE) [HTZT] |
| Gray-level Zone Size Matrices  (GLZSM) [9SAK] | GLSZM features quantify image textures by considering the frequency of occurrence of all isotone gray-level regions | Gray-level Non-Uniformity (GLN) [JNSA] Gray-level Variance (GLV) [BYLV] High Gray-level Zone Emphasis (HGZE) [5GN9] Large Zone Emphasis (LZE) [48P8] Large Zones High Gray-level Emphasis (LZHGE) [J17V] Large Zones Low Gray-level Emphasis (LZLGE) [YH51] Low Gray-level Zone Emphasis (LGZE) [XMSY] Short Zone Emphasis (SZE) [5QRC] Short Zones High Gray-level Emphasis (SZHGE) [HW1V] Short Zones Low Gray-level Emphasis (SZLGE) [5RAI] Zone Percentage (ZP) [P30P] Zone Size Non-Uniformity (ZSN) [4JP3] Zone Size Variance (ZSV) [3NSA] |
| Neighborhood Gray-Tone Difference Matrix  (NGTDM) [IPET] | NGTDM features quantify the difference between a voxel of interest and average of the surrounding 3 × 3 × 3 voxel neighborhood | Busyness (BUSY) [NQ30] Coarseness (COAR)* Complexity (CPLX) [HDEZ] Contrast (CONT) [65HE] Strength (STRG)* |
| Intensity-based Statistics  (IS) [UHIW] | Statistics of region of interest. | Kurtosis [IPH6] Skewness [KE2A] Variance [ECT3] |

*NGTDM-based coarseness and strength are calculated as defined by Amadasun and King^43^.

# Supplementary Figures


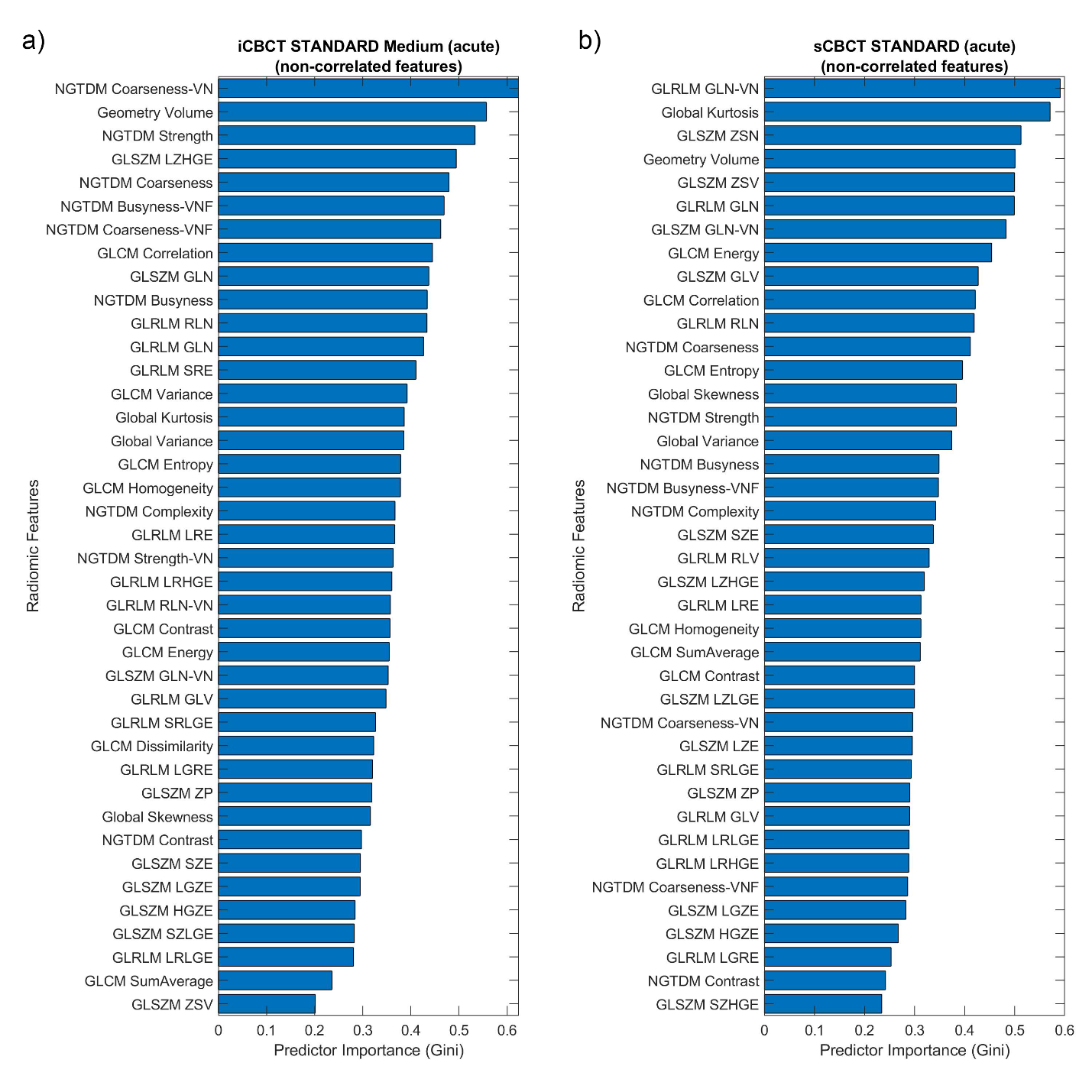


Supplemental Figure 1: The Acute GU toxicity final predictor importance ranking after removing the lower ranked correlated features for a) iCBCT-based and b) sCBCT-based DRF models.


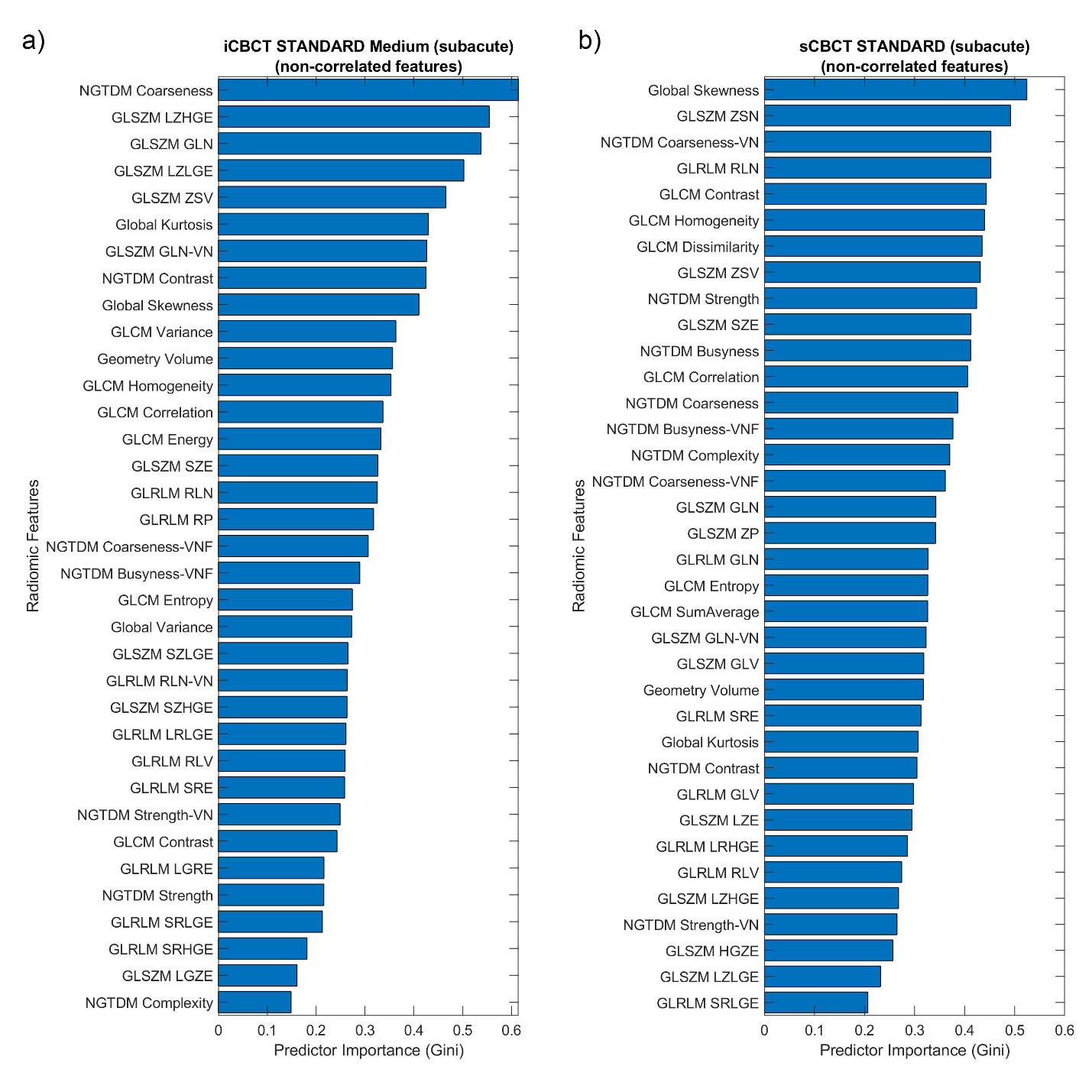


Supplemental Figure 2: The Sub-Acute GU toxicity final predictor importance ranking after removing the lower ranked correlated features for a) iCBCT-based and b) sCBCT-based DRF models.


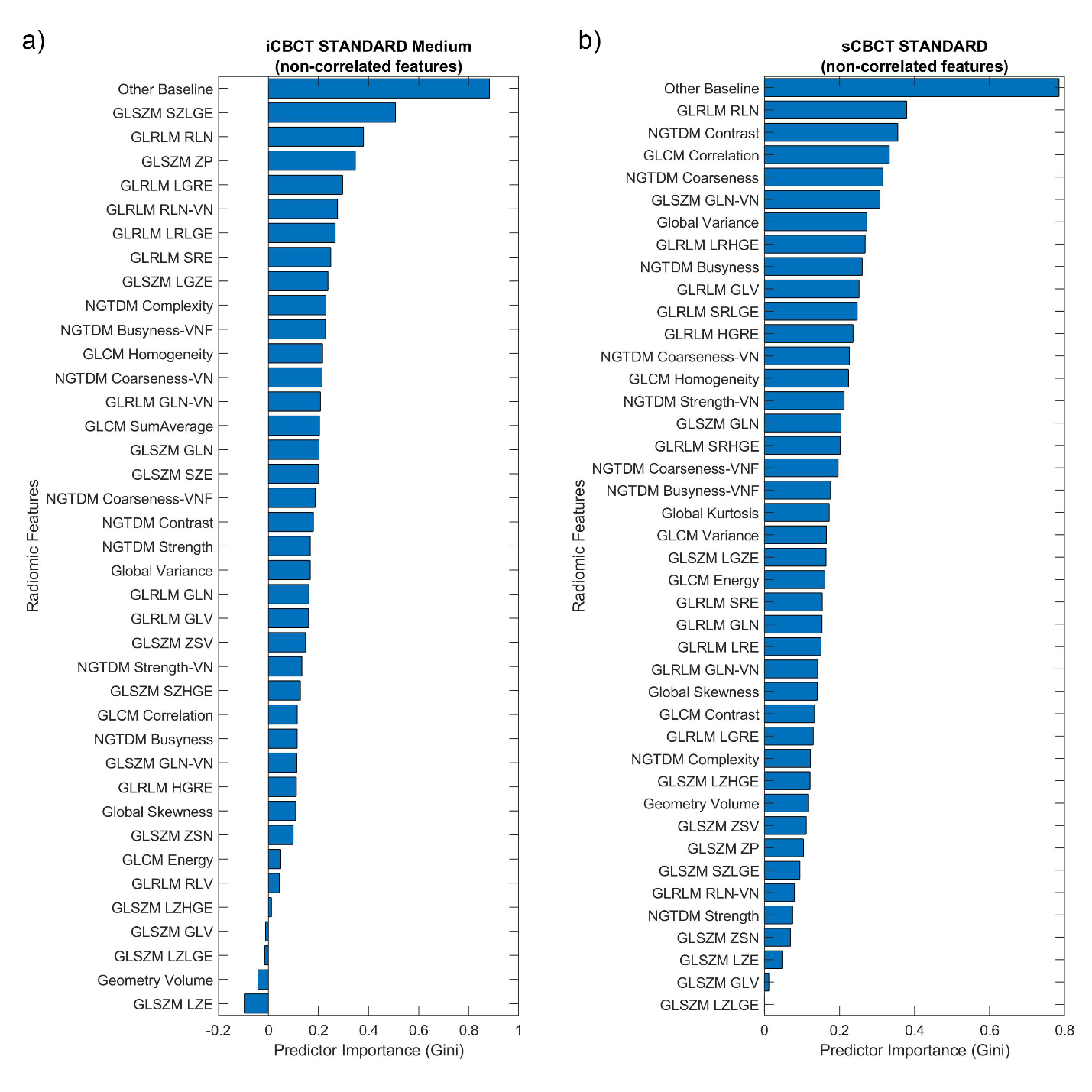


Supplemental Figure 3: The ∆IPSS GU toxicity final predictor importance ranking after removing the lower ranked correlated features for a) iCBCT-based and b) sCBCT-based DRF models.
